# High-Throughput Quantification of Bacterial-Cell Interactions Using Virtual Colony Counts

**DOI:** 10.3389/fcimb.2018.00043

**Published:** 2018-02-15

**Authors:** Stefanie Hoffmann, Steffi Walter, Anne-Kathrin Blume, Stephan Fuchs, Christiane Schmidt, Annemarie Scholz, Roman G. Gerlach

**Affiliations:** ^1^Project Group 5, Robert Koch Institute, Wernigerode, Germany; ^2^Department of Bioorganic Chemistry, Leibniz Institute of Plant Biochemistry, Halle, Germany; ^3^Division 13: Nosocomial Pathogens and Antibiotic Resistances, Robert Koch Institute, Wernigerode, Germany

**Keywords:** *Salmonella*, invasion, adhesion, intracellular replication, gentamicin protection assay, virtual colony count, bacterial quantification, cell culture infection model

## Abstract

The quantification of bacteria in cell culture infection models is of paramount importance for the characterization of host-pathogen interactions and pathogenicity factors involved. The standard to enumerate bacteria in these assays is plating of a dilution series on solid agar and counting of the resulting colony forming units (CFU). In contrast, the virtual colony count (VCC) method is a high-throughput compatible alternative with minimized manual input. Based on the recording of quantitative growth kinetics, VCC relates the time to reach a given absorbance threshold to the initial cell count using a series of calibration curves. Here, we adapted the VCC method using the model organism *Salmonella enterica* sv. Typhimurium (*S*. Typhimurium) in combination with established cell culture-based infection models. For HeLa infections, a direct side-by-side comparison showed a good correlation of VCC with CFU counting after plating. For MDCK cells and RAW macrophages we found that VCC reproduced the expected phenotypes of different *S*. Typhimurium mutants. Furthermore, we demonstrated the use of VCC to test the inhibition of *Salmonella* invasion by the probiotic *E. coli* strain Nissle 1917. Taken together, VCC provides a flexible, label-free, automation-compatible methodology to quantify bacteria in *in vitro* infection assays.

## Introduction

The ability of pathogenic bacteria to interact with eukaryotic cells depends on a complex interplay of bacteria- and host-derived factors. At the bacterial side the presence of virulence factors such as adhesins and secretion systems significantly contributes to this interaction (Gerlach and Hensel, 2007a). Cell culture based infection models have been used with great success to identify and characterize virulence factors important for the interaction with defined cell types. As a main readout these analyses rely on the enumeration of bacteria bound to or internalized within these cells. The gold standard to quantify bacteria in these assays is still plating of a dilution series on solid agar and counting of the resulting colony forming units (CFU). This method is laborious and inherits many manual steps making it hard to establish a standardized or automated procedure required for high-throughput analyses. Therefore, alternative methods based on radioactive, fluorescence, luminescence, or chromogenic labeling of bacteria have been developed (Acord et al., 2005; Vesterlund et al., 2005; Martens-Habbena and Sass, 2006). Labeling is achieved through either genetic modification or preparatory staining of the bacteria. Depending on the bacterial species or strain such manipulations or processing steps might not be possible or could exert unpredictable effects on cell physiology. Furthermore, specialized and sensitive detection equipment is required.

As a label-free alternative to CFU counting a method called “Virtual Colony Count” (VCC) has been developed (Brewster, 2003). The principle of VCC shows many similarities to quantitative polymerase chain reaction (qPCR). While in qPCR assays the increase in fluorescence intensity is monitored over time, VCC monitors quantitative growth kinetics based on absorbance measurements. Instead of cycles required to meet a certain fluorescence threshold (C_t_) in qPCR, VCC relies on the time to reach a given absorbance threshold (T_t_). Correlation of T_t_ to the initial cell count is achieved with the help of a series of calibration curves (Brewster, 2003). So far, use of VCC was limited to enumerate cells in pure bacterial cultures, e.g., to quantify the bactericidal effect of antimicrobial peptides (Ericksen et al., 2005; Xie et al., 2005; Zou et al., 2008; Rajabi et al., 2012; Zhao et al., 2012; Pazgier et al., 2013), but was not applied to infection models.

Pathogenicity of *Salmonella enterica* serovar Typhimurium (*S*. Typhimurium) has been characterized in detail with the help of cell culture-based infection models. In conjunction with animal models it could be demonstrated that virulence of *S*. Typhimurium is largely determined by a set of genes encoded on genomic loci called *Salmonella* pathogenicity islands (SPI) (Gerlach and Hensel, 2007b). The type three secretion systems (T3SS) encoded by SPI-1 and SPI-2 (T3SS-1/2) are *inter alia* required for trigger-like invasion of non-phagocytic cells and intracellular survival and replication, respectively (Fàbrega and Vila, 2013). For invasive pathogens such as *S*. Typhimurium, the gentamicin protection assay is an established *in vitro* methodology to distinguish intracellular from extracellular bacterial cells. While the latter ones are killed by antibiotic treatment, intracellular organisms are protected and survive. After subsequent lysis of host cells, bacteria are quantified to determine the invasiveness or their ability for intracellular replication (Devenish and Schiemann, 1981). Here we demonstrate a workflow, including automated data analysis, to apply VCC for bacterial quantification in gentamicin protection assays using three different *Salmonella* infection models.

## Materials and methods

### Bacterial strains and growth conditions

All bacterial strains used are listed in Table 1. *Salmonella* mutants are all isogenic to the wild-type strain *S*. Typhimurium NCTC 12023. Bacteria were grown aerated overnight (O/N) at 37°C in LB medium without or with the addition of 50 μg ml^−1^ kanamycin, where appropriate. For calibration curves, bacterial cultures were inoculated 1:100 in fresh LB and continued to grow aerated in a roller drum (TC-7, New Brunswick, Edison, NJ, USA) for 2 h 30 min at 37°C until an OD_600_ of 1.4–1.8 was reached. For invasion assays (HeLa, MDCK), bacterial cultures were inoculated 1:31 in fresh LB and continued to grow aerated in a roller drum for 3 h 30 min at 37°C.

**Table 1:** Bacterial strains used in this study.

**Strain**	**Relevant characteristic(s)**	**Source or reference**
***S. enterica*** **serovar Typhimurium strains**		
NCTC12023	Wild type (WT)	NCTC, Colindale, UK
MvP818	*invC* FRT (T3SS-1^−^)	Gerlach et al., 2008
WRG226	*sseJ::3xFlag* FRT, *ssaV* FRT (T3SS-2^−^)	Lab collection
WRG238	SiiF_E627Q_ (Walker B mutant, SPI-4^−^)	Lab collection
WRG300	*malYX*::P_EM7_ I-SceI *aph*, Kan^r^	Lab collection
*E. coli* strain
Ec^Nissle^	Nissle 1917 wild-type strain	Bärbel Stecher, Munich

### Cell culture

MDCK cells were cultured in MEM medium (Biowest, Nuaillé, France) supplemented with 10% FCS, 2 mM Glutamax (Thermo, Karlsruhe, Germany), non-essential amino acids (Biowest), 100 U ml^−1^ penicillin and 100 μg ml^−1^ streptomycin (Biowest). For invasion assays, cells were seeded at a density of 8 × 10^4^ per well in 96-well plates (Cellstar #655180, Greiner Bio-One, Germany) using the four inner rows. Cells were allowed to differentiate for 10–11 days. The growth medium was replaced by fresh medium every other day and was changed at least 4 h before infection after one washing step with PBS to complete cell culture medium without antibiotics. HeLa and RAW264.7 cells (LGC Standards, Wesel, Germany) were cultured in DMEM medium (high glucose, stable glutamine, sodium pyruvate) (Biowest) supplemented with 10% FCS. HeLa and RAW264.7 were seeded in 96-well plates (Greiner Bio-One) 24 h before infection using the four inner rows at a density of 6 × 10^3^ per well or 5 × 10^4^ per well, respectively. All cell lines were kept under a humidified atmosphere of 5% CO_2_ at 37°C.

### Infection and virtual colony count (VCC) assay

Overnight (RAW264.7) or sub-cultured (HeLa, MDCK) bacteria were adjusted to an OD_600_ of 0.2 (~2 × 10^8^ CFU ml^−1^) in sterile PBS. Bacteria were then diluted in complete cell culture medium without antibiotics to get the desired MOI and cells were infected with 100 μl of bacterial suspension per well. Infection was allowed for 25 min (MDCK, HeLa) or 60 min (RAW264.7) at 37°C. Non-adherent bacteria were removed by one washing step with pre-warmed PBS and cells were further incubated for 1 h with complete cell culture medium containing 100 μg ml^−1^ gentamicin to kill non-invaded, extracellular bacteria. For infection of RAW264.7 cells two plates were infected in parallel and cell culture medium containing 10 μg ml^−1^ gentamicin was used for the remainder of the experiment. After the indicated incubation period host cells were washed twice with PBS and lysed by the addition of 100 μl pre-warmed lysis buffer containing 2.0% (v/v) Elugent (#324707, Merck, Darmstadt, Germany), 0.0625% (v/v) Antifoam B emulsion (#A5757, Sigma-Aldrich, Steinheim, Germany) in PBS for 30 min shaking at 37°C. VCC was used to quantify intracellular bacteria. After complete lysis the four outer rows of the 96-well plate were filled with 100 μl per well of the inoculi, 10-fold diluted in lysis buffer. Microbial growth was initiated with the addition of 100 μl pre-warmed, 2-fold concentrated BHI medium to each well. The microtiter plate was incubated with lid under constant shaking at 37°C in a microplate reader (Infinite M1000, Tecan, Grödig, Austria) and absorbance at 600 nm was measured every 5 min. In case of HeLa infections additional serial dilutions of the lysates and inoculi were made in PBS and spot-plated on LB agar for CFU enumeration.

### Data analysis

The complete analysis pipeline was implemented in the statistical programming language “R” (R Core Team, 2017). A 5-parameter log-logistic fit (formula 1) implemented in the “R” package “drc” (Ritz et al., 2015) was applied to an analysis window of the raw data which range was defined by a fixed first data point and the position of the maximum slope or of the first local maximum minus a predefined fixed number of data points.

(1) $$y=c+\frac{d-c}{{\left(1+e^{b*\left(x-e\right)}\right)}^{f}}$$

Subsequently, T_t_ was defined as intersection point between the fitted 5-parameter log-logistic function and a line following this formula:

(2) y=0.02+c

Here, *c* represents the lower asymptote of the fitted log-logistic function. We provide two “R” scripts (Supplementary Data) including sample data files which share the methodology to determine T_t_ from growth curves and allow for further correlation to log(CFU) on the basis of calibration curves or calculation of VCC and invasion rates, respectively. Alternatively, the R package “chipPCR” (Rödiger et al., 2015) was used to analyze the raw data. With “chipPCR” a linear background function was calculated using the “least” method from a data window within the lag phase and subsequently subtracted from the raw data. The normalized growth curves were fit using the moving average (mova) function. For calculation of the time to reach the threshold of 0.02, the intersection with a linear regression of four data points near the threshold was used. Data was visualized using either the “R” package “ggplot2” (Wickham, 2016) or Prism v7.03 (GraphPad Software, San Diego, CA, USA).

## Results

### Curve fit and calculation of threshold times (T_t_)

A challenging aspect of VCC is the determination of the time to reach a certain absorbance threshold (T_t_). Previously, T_t_ was calculated from data normalized by subtracting the first (Ericksen et al., 2005) or the second (Rajabi et al., 2012; Zhao et al., 2012, 2013) data value from raw data or from a linear fit including five data points around an absorbance threshold (Brewster, 2003). In another approach growth curves with significant differences in the baseline and maximal absorbance were background-subtracted and similarly scaled based on the detection of their maximum slope (Brewster, 2003). Our goal was to establish a more reliable and empiric methodology for automatic calculation of T_t_ from relatively noisy data originating from host cell debris-containing samples. For that, robust background detection and fitting of the individual growth curves is required. We tested two different curve fitting and normalization methods using the statistical programming language “R” (R Core Team, 2017).

**Figure 1:**
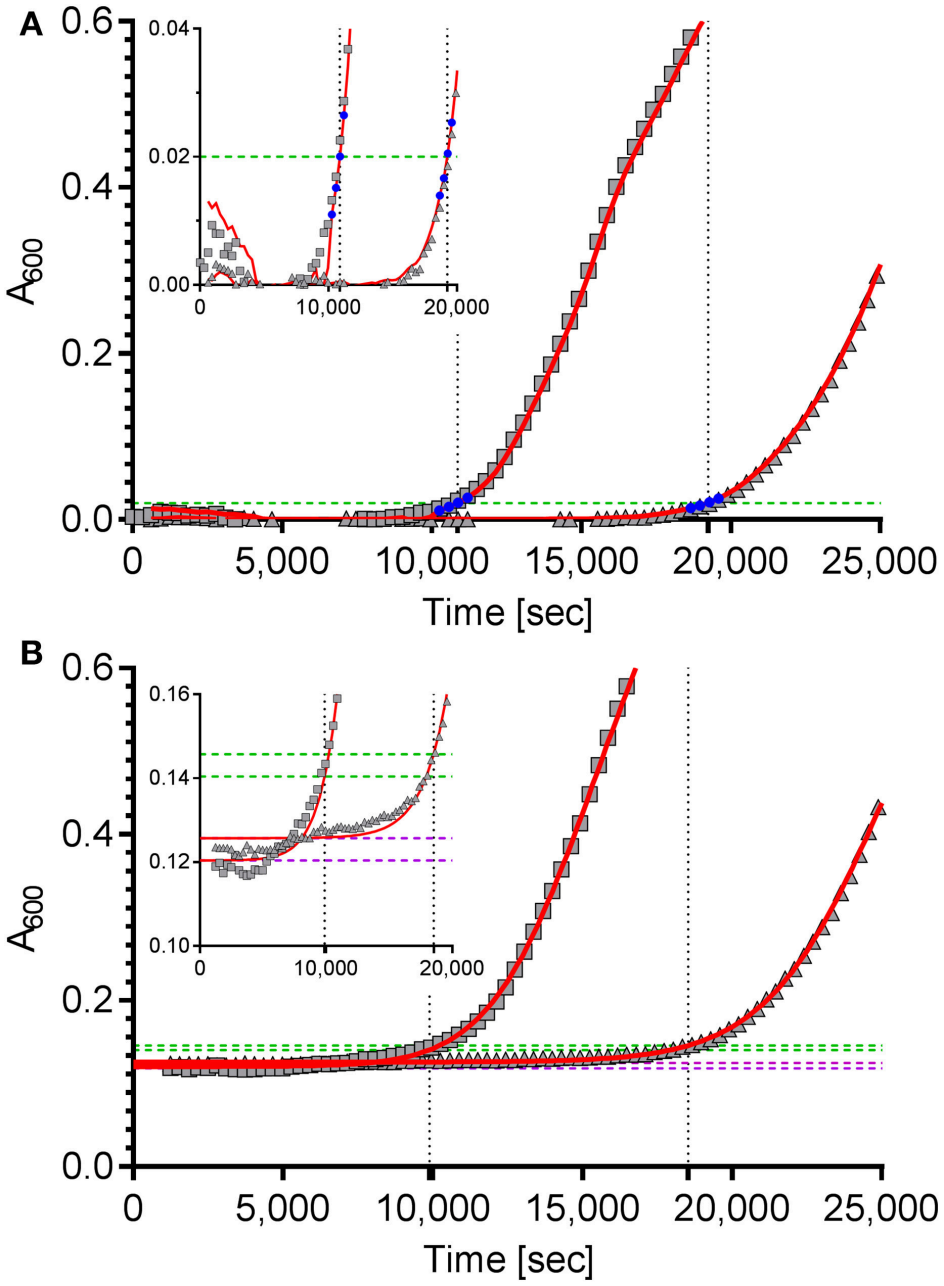
Calculation of the time to reach the threshold of ΔA_600_ = 0.02 of two example growth curves using two different curve fitting and normalization methods. **(A)** The “R” package “chipPCR” was used to analyze the raw data (gray squares and triangles). After subtraction of a background function, the curves were fit using the moving average (mova) function (red lines). To calculate the time to reach the threshold of 0.02 (dashed green line), the point of intersection with a linear regression of four data points (blue) near the threshold was used. Here, threshold times of 10,874 s and 19,243 s (dotted black lines) were determined. **(B)** The same raw data sets shown in **(A)** were fit to a 5-parameter log-logistic function implemented in the “R” package “drc” (red lines). The thresholds of ΔA_600_ = 0.02 were calculated individually for each data set (dashed green lines) by adding up 0.02 to the lower asymptotes (dashed purple lines) of the log-logistic functions. Threshold times of 9,890 s and 18,534 s were calculated from the intersection points (dotted black lines). Insets: zoomed views of the data ranges showing the transition to logarithmic growth.

The first approach is based on the package “chipPCR” (Rödiger et al., 2015) for “R”. Although “chipPCR” is primarily designed for the analysis of qPCR data, we utilized it successfully for the analysis of bacterial growth curves. ChipPCR is capable of calculating a linear background function from a data window within the lag phase. For the two example data sets shown in Figure 1A data points 10–30 were used for this purpose. The moving average (“mova”) curve fitting method was applied to the background-subtracted raw data. The threshold times T_t_ were calculated based on an absorbance threshold of 0.02 which was previously shown to be optimal for VCC (Ericksen et al., 2005). For that the intersection points of the absorbance threshold (Figure 1A, dashed green line) with a linear regression from four data points (Figure 1A, blue) derived from the “chipPCR” curve fit and surrounding A_600_ = 0.02 were calculated. Subsequently, T_t_ of 10,874 s and 19,243 s (Figure 1A, dotted black lines) were determined for the exemplary dataset.

For the second approach the “R” package “drc” (Ritz et al., 2015) was used which provides a set of model functions for dose-response analyses. We decided to use the 5-parameter log-logistic function included in “drc” because this model has been shown to fit bacterial growth curves very well (Zwietering et al., 1990) and was especially robust in background (lag phase) determination even with noisy data which is essential for exact T_t_ calculation (data not shown). Unfortunately, we frequently observed low quality fits in cases were stationary growth phase followed an inhomogeneous trend. As a consequence, a method for automatic determination of an analysis window only including the lag- and the logarithmic growth phases was implemented in our “R” script. While a fixed number of initial data points can be excluded to account for noise due to the possible presence of incompletely solubilized debris or air bubbles at the beginning of the kinetic, the end of the analysis window is individually determined for each growth curve. Depending on the analyzed data two methods can be accessed in the script to define the last data point to be included in the curve fit: by detecting (i) the maximum slope or (ii) the first local maximum. After the curve fit the lower asymptote of the log-logistic function was used as individual background reference (Figure 1B, dashed purple lines). Subsequently, an absorbance threshold of ΔA_600_ = 0.02 was set based on these references (Figure 1B, dashed green lines). Threshold times (T_t_) were calculated from the intersection points with the fitted logistic function (Figure 1B, dashed black lines). Using this method, T_t_ of 9,890 s and 18,534 s were determined for the two exemplary growth curves shown in Figure 1B, respectively.

The quality of curve fits produced by “chipPCR” was highly depending on the careful window selection for background calculation. Although “chipPCR” has the potential for superior curve fitting results (see Figure 1 insets) it required manual adjustments of parameters for each data set. In contrast, the log-logistic fit based on an analysis window calculated individually for each growth curve did not require any further adjustments. After establishing global parameters depending on the bacterial strain, growth medium and microplate reader used, we found the log-logistic fitting of growth data to be more consistent and robust for background estimation of rather noisy data. Therefore, the log-logistic fitting methodology was used in all further analyses.

### Optimization of host cell lysis

Efficient host cell lysis is of critical importance for the turbidimetric detection of bacterial growth. In classical gentamicin protection assays the detergent Triton X-100 is often used for cell lysis (Small et al., 1987). We observed that addition of Triton X-100 resulted in clumping of host cells (data not shown) that would have required further mechanical treatment (e.g., pipetting). These additional processing steps should be circumvented as a source of errors and to streamline batch processing. We found the detergent Elugent, a mixture of alkyl glycosides and inexpensive substitute for octyl glycoside, very effective to lyse host cells. With this detergent we were able to obtain efficient lysis of HeLa and RAW264.7 cells at concentrations of 0.5% (v/v) whereas for confluent MDCK cell layers a final concentration of 2.0% (v/v) was required (data not shown). The addition of the detergent introduced foaming which heavily interfered with optical detection of bacterial growth. To prevent foam formation of the lysis buffer the silicone-based emulsion “Antifoam B” was added. We tested different concentrations of Antifoam B and Elugent for their potential inhibition of bacterial growth which should be visible through a delay of T_t_ compared to controls. For Antifoam B, no significant impact on bacterial growth was observed for 1:200 or higher dilutions (Supplementary Figure 1A). Elugent concentrations of 0.125% (v/v) and above slightly attenuated bacterial growth (Supplementary Figure 1B). To ensure efficient lysis of all host cells a buffer containing 2.0% (v/v) Elugent in the presence of 0.0625% (v/v; 1:1,600 dilution) Antifoam B was used in all subsequent experiments. A control experiment revealed no significant difference in CFU counts comparing bacteria treated with 2% (v/v) Elugent or left untreated (Supplementary Figure 1C).

### Generation of calibration curves

VCC requires a set of calibration curves with known amounts of bacteria for absolute quantification of bacterial numbers. In an exemplary analysis a ten-fold dilution series was prepared from logarithmically growing *S*. Typhimurium in lysis buffer starting at 10^7^ bacteria per 100 μl down to 10^−3^ bacteria per 100 μl. By applying 100 μl to each well, a complete column of a 96-well plate was used for every dilution step. After addition of 100 μl 2-fold concentrated brain-heart infusion (BHI) broth, quantitative growth curves were recorded in a temperature-controlled microplate reader (Supplementary Figure 2A). In parallel, aliquots of the 10^2^ and 10^3^ dilutions were spotted onto LB agar plates for CFU counting. We provide a customized “R” script (“VCC_calibration.R,” Supplementary Data) which automates the calculation of T_t_ as described above and correlation of T_t_ against the logarithm of CFU counts. Growth was detected for seven out of eight wells at a concentration of 10 bacteria/well which can be defined as the detection limit of the method. After excluding the single data point for ~1 bacteria per well (10^0^, column 8) from the data set shown in Supplementary Figure 2A, linear regression could be calculated with a coefficient of determination (*R*^2^) of 0.99612 (Supplementary Figure 2B). Furthermore, the script output contains a residuals vs. fitted plot (not shown) and additional graphical representations of T_t_ and the residual standard error of the non-linear fit (S) in a 96-well plate layout (Supplementary Figure 2C) as well as graphs of the log-logistic fit of each growth curve and a summary in text format (data not shown). Notably, the parameters of the calibration curves will vary depending on the bacterial strain and the incubation conditions (medium, microplate reader, temperature, shaking etc.) used and need to be determined individually. Moreover, for exact calculation of bacterial numbers in cell culture infection models the generation of separate calibration curves in the presence of host cells is required.

### Comparative analysis of *Salmonella* invasion in HeLa cells

**Figure 2:**
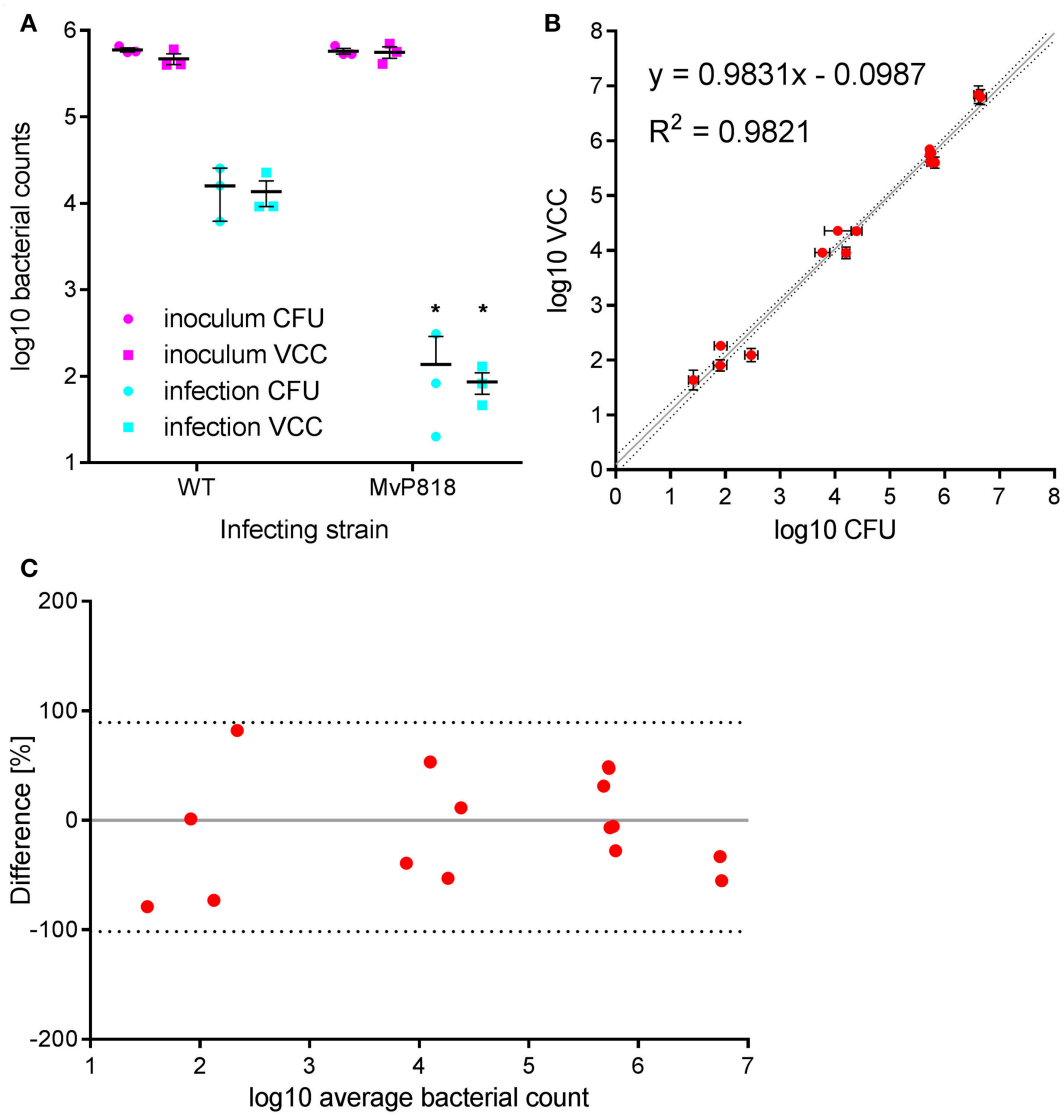
*Salmonella* invasion of HeLa cells was used to evaluate VCC against CFU counting on solid agar in a direct side-by-side comparison. **(A)** Bacterial numbers in the inoculum (magenta) and intracellular bacteria recovered after 1 h of infection (cyan) with wild-type (WT) or a T3SS-1 deficient *Salmonella* strain (MvP818) at an MOI of 100 from three independent experiments done in triplicates including means and SD are depicted. Statistical analysis by Student's *t*-test demonstrates that mutant MvP818 is highly attenuated compared to WT using both enumeration methods. ^*^P < 0.05 **(B)** Samples from four independent experiments enumerated with both methods are shown with *SD* indicated separately for VCC and CFU counting. The coefficient of determination (*R*^2^) is given for the correlation of both methods. Dotted black lines indicate the 95% confidence interval of the linear regression (gray line). **(C)** The data shown in **(B)** were plotted in a Bland-Altman diagram. Here, the mean of the corresponding bacterial counts determined with both methods was used as reference (0, gray line). The red dots represent the relative difference of the CFU counts compared to VCC by calculating 100^*^(CFU-VCC)/average. The 95% limits of agreement (dashed black lines) are at −101.6 and 89.4%, respectively.

HeLa cells are widely used as a well-characterized infection model to investigate invasion of *Salmonella* in non-phagocytic cells. *S. enterica* can trigger its own uptake in these cells with the help of effector proteins translocated by the T3SS encoded on SPI-1 (Cain et al., 2008). *Salmonella* invasion of HeLa cells was used to evaluate VCC against CFU counting on solid agar in a direct side-by-side comparison. Besides wild-type (WT) bacteria the invasion-deficient *S*. Typhimurium mutant MvP818, which lacks the T3SS-1 ATPase InvC, was used in this infection model. For the two quantification methods two 96-well plates with HeLa cells were infected in parallel from the same inoculi at a multiplicity of infection (MOI) of 100. Serial dilutions of the inoculi were made in PBS and spotted onto LB agar plates for CFU counting. For VCC the inoculi were diluted 1:10 in lysis buffer, stored at 4°C and warmed to 37°C 10 min before bacterial growth was initiated with the addition of 2x BHI. After a standard gentamicin protection assay, lysis buffer was added to liberate intracellular bacteria from the host cells. In case of VCC, bacterial growth was started by adding 100 μl of 2x BHI. For CFU counting serial dilutions of the lysed host cell samples were spotted onto LB agar plates. To calculate VCC from T_t_ we used two different calibration curves generated in the absence (inoculi) or in the presence (intracellular bacteria) of HeLa cells. A customized “R” script is provided (“VCC_invasion.R,” Supplementary Data) where the parameters of both calibrations curves can be entered as variables and which can automate the determination of T_t_ with subsequent conversion to VCC and calculation of invasion rates (normalized to the respective inoculi). In Figure 2A the bacterial numbers in the inoculum and of intracellular bacteria recovered 1 h after infection are depicted. As expected, the mutant MvP818 is highly attenuated for invasion compared to WT using both quantification methods. Although both methods yielded very comparable results in terms of absolute numbers, VCC showed lower variances for intracellular bacteria. There was an adequate correlation (*R*^2^ = 0.9821) of both detection methods in four independent experiments (Figure 2B). A Bland-Altman diagram of the same data revealed an almost unbiased ~2-fold variation from the average of both methods (Figure 2C). In conclusion, our comparative analysis of HeLa infections showed that VCC and the standard CFU counting produced very similar results.

### Infection of polarized MDCK cells

**Figure 3:**
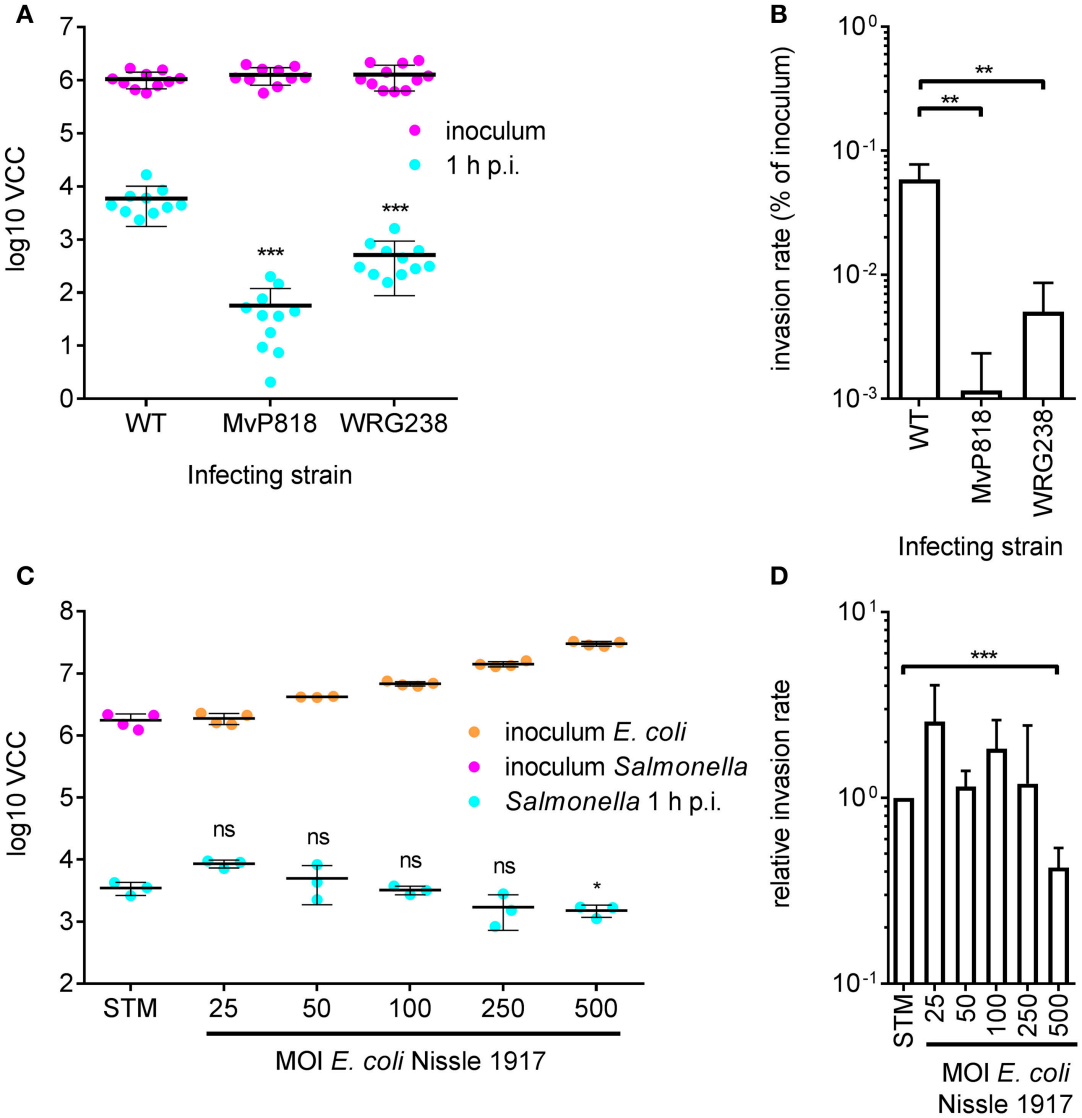
Quantification of invasion of polarized MDCK cells with VCC. **(A)** Bacterial numbers in the inoculum (magenta) and intracellular bacteria recovered from MDCK infected with the indicated *Salmonella* strains at an MOI of 25 1 h post-infection (p.i.) (cyan) from three independent experiments done in 4-fold replicates are depicted. **(B)** From the data shown in **(A)** the invasion rates of the strains were calculated. Both mutants showed a significantly reduced invasiveness compared to the WT. **(C)** Cells were incubated for 4 h before infection using different MOIs of the probiotic *E. coli* strain Nissle 1917 (Ec^Nissle^) as indicated. The amounts of Ec^Nissle^ cells were determined for each MOI using VCC (orange dots). MDCK were subsequently infected with a kanamycin resistant *S*. Typhimurium WT strain (STM) at an MOI of 25 (magenta) as described in **(A)**. Intracellular *Salmonella* (cyan) were significantly reduced when cells were pre-incubated with Ec^Nissle^ at an MOI of 500. Means and standard deviations from one representative out of three similar experiments done in 4-fold replicates (dots) are depicted. **(D)** The relative invasion rates of STM depending on the MOI of Ec^Nissle^, normalized to controls without Ec^Nissle^ (STM), were calculated from data of three independent biological replicates. Statistical analysis by Student's *t*-test was done by comparing to WT or individual strains as depicted: ^***^P < 0.001 ^**^P < 0.01; ^*^P < 0.05.

For efficient invasion of polarized epithelial cells such as Madin-Darby Canine Kidney (MDCK) cells, the functional cooperation of two *Salmonella* secretion systems is required. The *Salmonella* pathogenicity island 4 (SPI-4) encoded giant adhesin SiiE and cognate type 1 secretion system (T1SS) are needed to mediate efficient binding to the apical cell side. This intimate bacterial contact enables subsequent invasion using the T3SS-1 (Gerlach et al., 2008). MDCK cells were grown as a dense monolayer and hereinafter infected with *S*. Typhimurium WT as well as isogenic mutants deficient for a functional T3SS-1 (MvP818) or a SPI-4 T1SS (WRG238) at an MOI of 25. In WRG238 a chromosomal point mutation within the Walker B box of the ABC protein SiiF (E627Q) renders the T1SS non-functional. While VCC of the inoculi showed equal amounts for all strains, MvP818 and WRG238 were both attenuated for invasion. As expected, lower counts of intracellular bacteria were observed for MvP818 compared to the SPI-4 deficient WRG238 (Figure 3A). From these data the invasion rate in percent of the inoculum was calculated for each strain which confirmed our initial observations (Figure 3B). Although confluent MDCK cell layers are challenging to lyse our results showed that VCC can be successfully used for bacterial quantification in infection models based on polarized cells.

### Quantification of probiotic activity

We speculated that VCC could be also useful to analyze co-infection experiments when antibiotic markers are utilized to differentiate between the individual strains. As a proof of principle we chose to quantify probiotic activity. The probiotic *E. coli* strain Nissle 1917 (Ec^Nissle^) (Grozdanov et al., 2004) was shown to inhibit *Salmonella* invasion of INT407 (Altenhoefer et al., 2004). We wanted to test whether Ec^Nissle^ can exert its protective effect also in combination with polarized MDCK cells. MDCK were pre-incubated for 4 h with different amounts of Ec^Nissle^ corresponding to MOIs of 25 to 500. The numbers of *E. coli* cells used for this incubation step were confirmed by VCC (Figure 3C, orange dots). After removing unbound *E. coli*, the MDCK cells were infected with a kanamycin-resistant *S*. Typhimurium at an MOI of 25 as described above. Host cells were lysed and growth of intracellular *S*. Typhimurium was selectively started with addition of 2x BHI containing 50 μg ml^−1^ kanamycin. We did not observe growth of Ec^Nissle^ under these conditions (data not shown). Compared to untreated controls a significant reduction of intracellular *Salmonella* was evident with Ec^Nissle^ pre-incubation at MOI of 500 (Figure 3D). The observed dose-dependent probiotic activity of Ec^Nissle^ demonstrates that VCC can be used to analyze individual bacterial strains of a mixed population utilizing differences in antibiotic resistance phenotypes.

### Intracellular replication in macrophages

**Figure 4:**
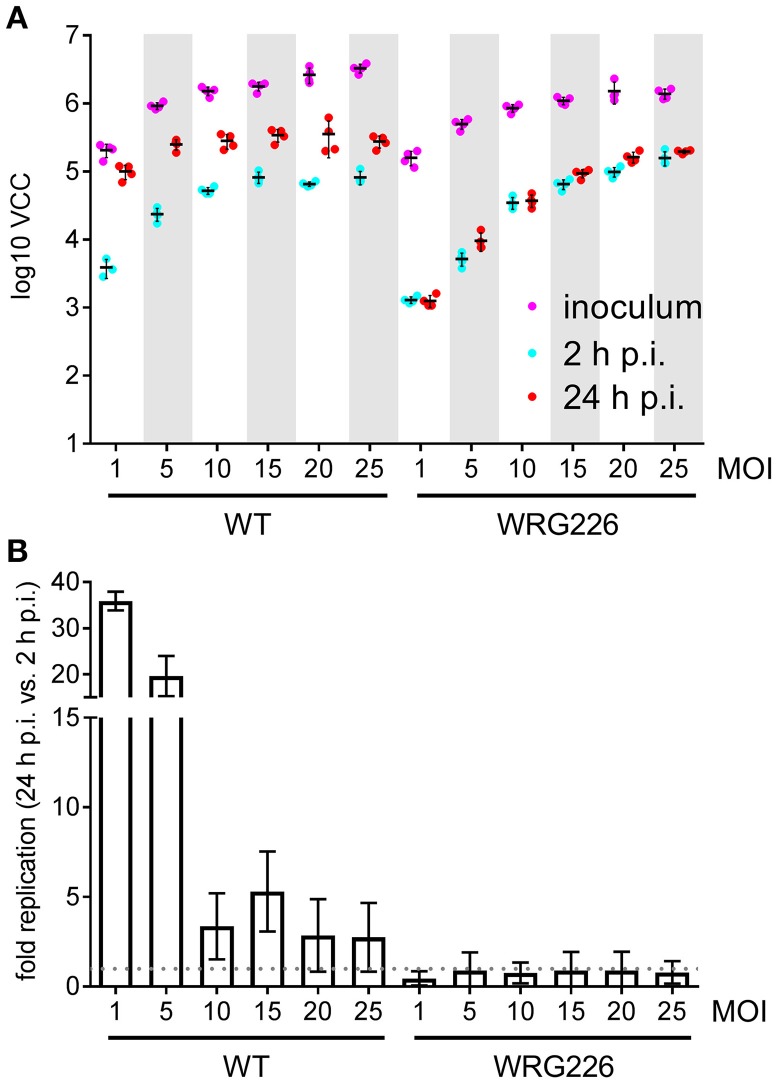
Replication of *Salmonella* within RAW264.7 macrophages. **(A)** Two 96-well plates with RAW 264.7 macrophages were infected in parallel with the strains and MOIs as indicated. In the first plate, bacteria in the inoculum (magenta) and intracellular bacteria after 2 h post-infection (p.i.) (cyan) were determined by VCC. The second plate was used for enumeration of intracellular bacteria after 24 h of infection (red). Means and standard deviations from one representative out of three similar experiments done in 4-fold replicates (dots) are depicted. **(B)** From the data of three independent biological replicates the fold replication of the strains were calculated. The maximum capacity of intracellular WT bacteria to replicate is reached after 24 h with an MOI of 1. The T3SS-2 deficient strain WRG226 showed no net replication (dashed gray line).

Intracellular survival and replication are key virulence capabilities of *S*. Typhimurium which can be assessed *in vitro* using RAW264.7 macrophages (Govoni et al., 1999). RAW264.7 cells were infected with *S*. Typhimurium WT and the isogenic mutant strain WRG226 which harbors a non-functional T3SS-2 as a negative control. Effectors of the T3SS-2 are essential for intracellular survival and replication of *Salmonella* (Hensel et al., 1998; Figueira and Holden, 2012). Two 96-well plates were infected in parallel with both strains using the MOIs as indicated in Figure 4. Bacteria in the inoculum and intracellular bacteria after 2 h of infection were determined by VCC using the first plate. The second plate was used for quantification of intracellular bacteria after 24 h of infection (Figure 4A). As intended, an increasing amount of bacteria in the inoculum could be detected with the increase in MOI. After 2 h the amount of intracellular bacteria increased with the MOI for WRG226 whereas uptake of WT bacteria reached a plateau with MOI of 15. After 24 h of infection, an increase of intracellular bacteria could be observed in case of WT where the maximum total amount was reached with an MOI of 5. In contrast, the T3SS-2 deficient strain WRG226 showed no increase in intracellular bacteria after 24 h (Figure 4A). By normalizing the 24 h time points with the 2 h values, the replication capability of the strains could be evaluated. WT bacteria exhibited a ~35-fold replication at a MOI of 1 which decreased to ~3-fold at a MOI of 25 (Figure 4B). In agreement with the pivotal role of T3SS-2 for intracellular replication in macrophages (Hensel et al., 1998), WRG226 showed no net increase of bacterial cells after 24 h (Figure 4B).

## Discussion

We could establish VCC as a robust method for bacterial enumeration in three different cell culture infection models using the bacterial pathogen *S*. Typhimurium. A hallmark of *Salmonella* virulence is its ability for trigger-like invasion of non-phagocytic cells through T3SS-1 activity. Although T3SS-1 independent, zipper-like invasion mechanisms are described for *S. enterica* (Rosselin et al., 2011), its pivotal role is exemplified by highly reduced numbers of intracellular bacteria *in vitro* with non-functional T3SS-1 (Finlay et al., 1988; Galán and Curtiss, 1989). We reproduced this expected phenotype in HeLa and MDCK cells using VCC where a T3SS-1 deficient strain showed ~100-fold reduced invasion rates compared to WT controls. Efficient adhesion of *S*. Typhimurium to MDCK and other polarized cells is mediated by the SPI-4 encoded giant adhesin SiiE (Gerlach et al., 2007). As a consequence, invasion through T3SS-1 in these host cells is highly attenuated for SPI-4 deficient strains (Gerlach et al., 2008). In this regard our VCC data showed ~30-fold less SPI-4 negative bacteria compared to WT controls. A functional T3SS-2, however, is required to establish a replicative niche for intracellular *Salmonella* (Hensel et al., 1998; Fàbrega and Vila, 2013). Its crucial role was reproduced with VCC in RAW264.7 macrophage-like cells where the T3SS-2 mutant showed almost no net replication after 24 h of infection. These results are in good agreement with data from J774.1 macrophages using CFU counting (Hensel et al., 1998). Interestingly, the maximum uptake of WT bacteria was reached after 2 h at an MOI of 15. After 24 h the maximal number of intracellular bacteria was already reached with an MOI of 5. These results likely illustrate the limits of RAW264.7 cells in their capacity to take up and to support growth of intracellular bacteria under these experimental conditions.

In a further approach we tested the suitability of VCC to analyze co-infection experiments with an antibiotic marker to differentiate between probiotic Ec^Nissle^ and *Salmonella*. Supplementation of the growth medium with kanamycin allowed for selective growth of *S*. Typhimurium while no replication of Ec^Nissle^ was observed. Although only one bacterial strain can be quantified by VCC from one well, analysis of several replicate wells with the addition of strain-specific antibiotics would allow for high throughput competitive index assays (Segura et al., 2004).

Label free detection methods, such as thermography (Salaimeh et al., 2011) rely solely on bacterial growth. Here, the logarithmic signal amplification enables high sensitivity together with a huge detection range. In case of VCC we could quantify bacteria over six orders of magnitude with a detection limit of about 10 *S*. Typhimurium cells per well. In a direct comparison between CFU counting and VCC, bacterial numbers were within a 2-fold difference from the mean of both methods. Although this is a good correlation, it is known that quantification via CFU on agar plates has a certain inaccuracy (Naghili et al., 2013) In that light it is not optimal that VCC calibration curves are correlated to actual “colony counts” using CFU counting.

With optimized protocols classical CFU counting can be scaled up to medium-high throughput by doing serial dilutions in 96-well plates and multichannel pipetting on solid agar (Steele-Mortimer, 2008). With VCC similar or higher throughput is possible with the advantage that no dilution steps are required and data collection and analysis can be easily automated requiring only absorbance detection equipment and a freely available open source software. Given the benefits of VCC over CFU counting also some limitations of the technique should be considered. First, on agar plates differences in growth rates have an impact on colony size, but not on actual counts. However, the same differences very strongly affect the duration of the lag phase thereby leading to incorrect VCCs. Mutations or environmental changes (e.g., from intracellular to extracellular) might result in altered growth rates of bacteria. Based on these observations it is of utmost importance, that growth rates of test strains are comparable to those used in calibration curves. If this cannot be guaranteed separate calibration curves need to be recorded for the strains in question. Second, alterations in bacterial physiology with impact on absorbance measurements (e.g., apparent or real cell size, light scattering or biofilm formation) will interfere with VCC. These problems might not be easy to address but optimizations should include review of growth conditions (e.g., shaking speed, plate material) and careful correlation of T_t_ to log(CFU) in calibration curves.

The reliable, reproducible and batch-processing compatible calculation of T_t_ from raw absorbance data had to be established before VCC could be applied in infection models. This was especially challenging because initially incomplete lysis of host cells followed by the occurrence of debris results in relatively noisy raw data. Improved host cell lysis and homogenization might be achieved with alternative well geometries (Funke et al., 2009). However, to our knowledge no cell culture treated microtiter plates with other than round wells are available. As a solution we established a workflow based on a curve fit to the log-logistic distribution which is a robust and proven model of microbial growth (Zwietering et al., 1990) and enables largely automatic calculation of T_t_ from raw absorbance data using our customized “R” script.

In conclusion, VCC is a label-free, automation-compatible methodology suitable for enumeration of intracellular bacteria in *in vitro* infection models. This approach should be also coextensive with studying other types of bacterial-cell interactions such as adhesion. VCC is flexible and can be adapted to be used together with bacteria other than *Salmonella* and any type of temperature-controlled, shaking microplate reader after a few optimizations. With its high reproducibility and throughput, the method is especially qualified to foster the further characterization of the host-pathogen interface.

## Author contributions

SH, SW, CS, and AS performed experiments. SH, RG, A-KB, and SF analyzed the data. RG conceived the study and wrote the paper.

### Conflict of interest statement

The authors declare that the research was conducted in the absence of any commercial or financial relationships that could be construed as a potential conflict of interest.
