# Supplementary material for: High-Throughput Quantification of Bacterial-Cell Interactions Using Virtual Colony Counts
Source: Front Cell Infect Microbiol. 2018 Feb 15;8:43. doi: 10.3389/fcimb.2018.00043 (PMC5818393; doi:10.3389/fcimb.2018.00043)
Supplement: Supplementary file 1 [file DataSheet1.DOCX]

Supplementary Material

High-throughput quantification of bacterial-cell interactions using virtual colony counts

Stefanie Hoffmann^†^, Steffi Walter^†^, Anne-Kathrin Blume, Stephan Fuchs, Christiane Schmidt, Annemarie Scholz and Roman G. Gerlach*

*** Correspondence:** Corresponding Author: GerlachR@rki.de

^†^ These authors have contributed equally to this work.

# Supplementary Data

Two ‘R’ scripts are provided as supplementary data:

‘VCC_calibration.R’ – allows for correlation to log(CFU) on the basis of calibration curves, corresponding example data set: ‘example_calibration_curves.xlsx’

‘VCC_invasion.R’ – allows for calculation of VCC and invasion rates, corresponding example data set: ‘example_invasion.txt’

# Supplementary Figures and Tables

## Supplementary Figures


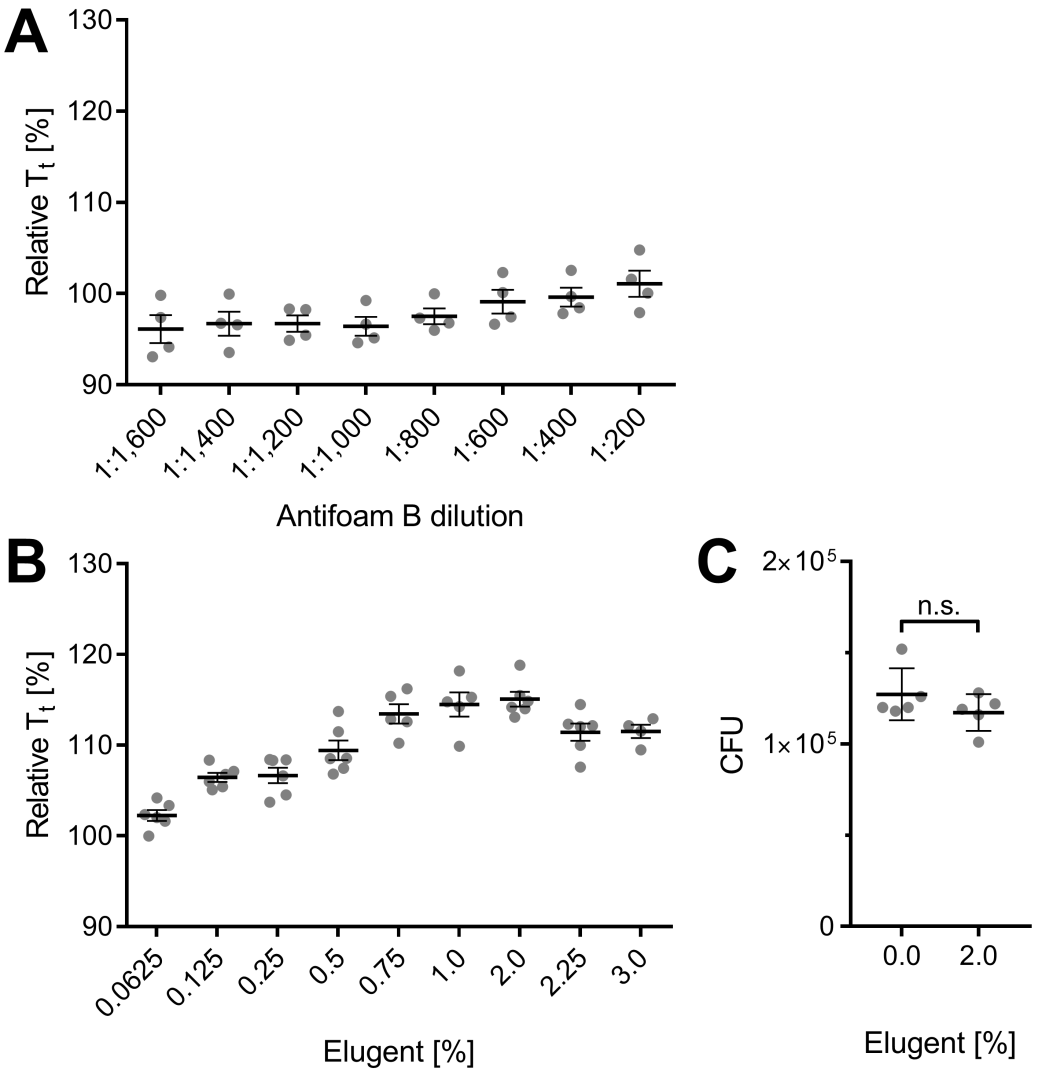


**Supplementary Figure 1.** The impact of different concentrations of Antifoam B and the detergent Elugent on the time to reach the threshold (T_t_). **(A)** Equal numbers of bacteria were added to BHI medium containing 0.5x PBS and the indicated dilutions of Antifoam B. Growth curves were fit using a 5-parameter log-logistic function in ‘R’. T_t_ was calculated and subsequently normalized to controls without Antifoam B (=100%). No significant influence on bacterial growth was observed for 1:200 or higher dilutions. **(B)** Using the same experimental setup as described in (A), relative T_t_ were determined for growth in medium containing different amounts of Elugent as indicated. To prevent foaming Antifoam B was added at a concentration of 0.0625% (1:1,600) to all samples. A slight attenuation in bacterial growth was observed for concentrations of 0.125% (v/v) Elugent and above. (**C**) An equal number (~1.2 × 10^5^) of bacteria was incubated with 2% (v/v) Elugent or left untreated. Subsequent CFU counting revealed no significant detrimental effect of Elugent on CFU counts.


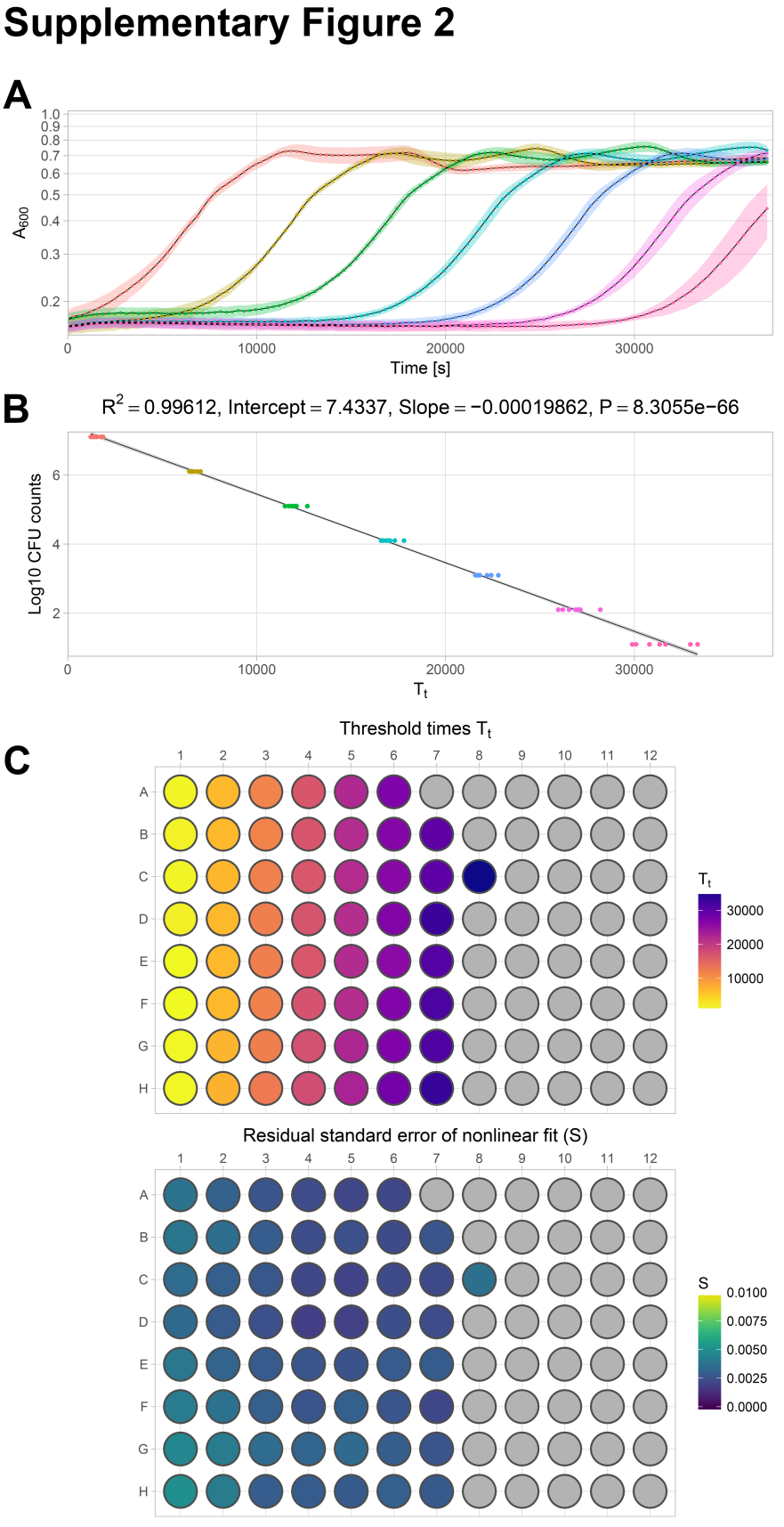


**Supplementary Figure 2.** Calibration curve to correlate the time to reach the threshold of ΔA_600_ = 0.02 (T_t_) to log(CFU). In **(A)**, mean (black lines) and SD (shaded areas) of growth curves of a dilution series of 10^7^ to 10^1^ CFU well^-1^ *S*. Typhimurium in lysis buffer/BHI done in 8-fold replicates are shown. **(B)** Parallel determination of colony-forming units (CFU) on agar plates allowed for correlation with threshold times (T_t_, dots) which were calculated as described in the text from the data shown in (a). The coefficient of determination (R^2^) and the 95 % confidence interval (grey area) is given for the linear regression (black line). **(C)** Schematic 96-well plate layout of the threshold times T_t_ (upper panel) and the residual standard errors of the nonlinear fit S (lower panel) calculated for each individual growth curve of the data set shown in (A).
